# Characterization of the transcriptome of an ecologically important avian species, the Vinous-throated Parrotbill *Paradoxornis webbianus bulomachus* (Paradoxornithidae; Aves)

**DOI:** 10.1186/1471-2164-13-149

**Published:** 2012-04-24

**Authors:** Jui-Hua Chu, Rong-Chien Lin, Chia-Fen Yeh, Yu-Cheng Hsu, Shou-Hsien Li

**Affiliations:** 1 Department of Life Science, National Taiwan Normal University, Taipei, 116, Taiwan; 2 Department of Natural Resources and Environmental Studies, National Dong Hwa University, Hualien, 97401, Taiwan

## Abstract

**Background:**

Adaptive divergence driven by environmental heterogeneity has long been a fascinating topic in ecology and evolutionary biology. The study of the genetic basis of adaptive divergence has, however, been greatly hampered by a lack of genomic information. The recent development of transcriptome sequencing provides an unprecedented opportunity to generate large amounts of genomic data for detailed investigations of the genetics of adaptive divergence in non-model organisms. Herein, we used the Illumina sequencing platform to sequence the transcriptome of brain and liver tissues from a single individual of the Vinous-throated Parrotbill, *Paradoxornis webbianus bulomachus*, an ecologically important avian species in Taiwan with a wide elevational range of sea level to 3100 m.

**Results:**

Our 10.1 Gbp of sequences were first assembled based on Zebra Finch (*Taeniopygia guttata*) and chicken (*Gallus gallus*) RNA references. The remaining reads were then *de novo* assembled. After filtering out contigs with low coverage (<10X), we retained 67,791 of 487,336 contigs, which covered approximately 5.3% of the *P*. *w*. *bulomachus* genome. Of 7,779 contigs retained for a top-hit species distribution analysis, the majority (about 86%) were matched to known Zebra Finch and chicken transcripts. We also annotated 6,365 contigs to gene ontology (GO) terms: in total, 122 GO-slim terms were assigned, including biological process (41%), molecular function (32%), and cellular component (27%). Many potential genetic markers for future adaptive genomic studies were also identified: 8,589 single nucleotide polymorphisms, 1,344 simple sequence repeats and 109 candidate genes that might be involved in elevational or climate adaptation.

**Conclusions:**

Our study shows that transcriptome data can serve as a rich genetic resource, even for a single run of short-read sequencing from a single individual of a non-model species. This is the first study providing transcriptomic information for species in the avian superfamily Sylvioidea, which comprises more than 1,000 species. Our data can be used to study adaptive divergence in heterogeneous environments and investigate other important ecological and evolutionary questions in parrotbills from different populations and even in other species in the Sylvioidea.

## Background

Adaptive divergence, driven by local adaptation to heterogeneous environments, has long been a fascinating topic in ecology and evolutionary biology [1]. Local adaptation not only leads to adaptive divergence between populations, but may also drive the process of speciation, i.e., ecological speciation [2,3]. It is often difficult to determine the extent of genetic differences associated with divergent selection. However, recent advances in genomic approaches such as genome scans and targeted resequencing provide illuminating ways to discover genes and genomic regions that might be involved in divergent selection [4]. For example, using genome-wide scans, genes that might be responsible for adaptation to high-elevation hypoxia were found in human Tibetan populations [5]. In addition, genes that might be associated with adaptation to serpentine soil have also been identified by genomic sequencing at the population level in the plant *Arabidopsis lyrata*[6]. Moreover, targeted resequencing in a specific chromosome region implied a selective target locus between diversifying ecotypes (M and S forms) of the African malaria mosquito *Anopheles gambiae*[7]. Those studies provide fresh insights into the genetic basis of adaptive evolution. However, genomic approaches are often applicable only to human or other model organisms for which genomic information is available. Although the cost of *de novo* genomic sequencing is much reduced by innovative sequencing technologies (e.g., Next Generation Sequencing, NGS, [8,9]), it is still out of the reach of studies of most non-model organisms which collectively represent the diversity and complexity of life forms.

To sequence a subset of the genome of a non-model organism, for example, the transcribed DNA within a given set of tissues under specific developmental stages or environmental conditions (transcriptome) may be a good alternative approach. Sequences of the transcriptome were used to develop polymorphic markers, such as single nucleotide polymorphisms (SNPs) and simple sequence repeats (SSRs), e.g. [10,11]. Because markers developed from the transcriptome are associated with expressed genes, they may better reveal intra- and inter-populational functional variations and are therefore suitable for studies of adaptive diversification or local adaptation [4]. Recent developments in RNA sequencing technology (RNA-Seq; reviewed in [12]), which take advantage of the deep sequencing power of NGS (see reviews in [13,14]) and efficient bioinformatic computations [15-17], have greatly enhanced the process of transcriptome characterization, and have turned transcriptome sequences into promising genetic resources for non-model organisms.

Herein, we report the characterization of the transcriptome of an ecologically important avian species in East Asia, the Vinous-throated Parrotbill (*Paradoxornis webbianus*, Timaliidae). The Vinous-throated Parrotbill is one of the most widely distributed resident avian species in East Asia [18]. It has a broad latitudinal and elevational distribution covering geographic ranges from northern Indochina to southern Siberia and from the eastern edge of the Tibetan Plateau to coastal China and the island of Taiwan. It occupies various types of habitats in China, while its island subspecies (*P. w. bulomachus*) is also considered to occupy the widest niche of any bird species in Taiwan with an extraordinary elevational distribution from sea level to 3100 m [18,19]. A steep elevational gradient can lead to high environmental heterogeneity (e.g., wide ranges of temperature and partial pressure of oxygen) within a short horizontal distance, which is considered a ‘natural laboratory’ with many resources that can be used to examine adaptive responses of populations to different environmental contexts [20]. The wide elevational distribution of *P. w. bulomachus* may provide a good system for addressing how divergent selection pressures posed by a steep elevational gradient may drive adaptive differentiation between populations living at different elevations.

In this study, we attempted to characterize the transcriptome sequenced from a single individual of *P. w. bulomachus* and explored its potential utility, for example, in developing genetic markers for use in future adaptive genetic studies. Specifically, we assembled and annotated transcriptome from Illumina short-read sequences and assigned them to different gene ontology (GO) categories. Putative genetic markers such as SNPs and SSRs were also identified from the transcriptome. Despite only sequencing a single individual’s transcriptome, SNPs were identified through heterozygous sites of the assembled contigs. We then identified genes in our transcriptome database which also appeared in an *a priori* list of candidate genes that might respond to hypoxia [5] and be involved in climate-related adaptation [21], and matched them to specific GO terms associated with environmental adaptation. Sequences of these genes can be used to design targeted gene-resequencing experiments to detect the genetic signature of elevational adaptation in the Vinous-throated Parrotbill. Our results demonstrate that transcriptome sequences, even from a single individual, can serve as a rich genetic resource to investigate ecological and evolutionary processes.

## Results and discussion

### Transcriptome assembly

We received approximately 10.1 × 10^9^ base pairs (Gbp) of sequence data, composed of 119,408,529 reads with an average length of 84.6 bp. Base call accuracy for most of the reads (92.6%) reached a threshold of 99% (i.e., the probability of a base call error of ≤ 0.01; Q20). In total, 487,336 contigs were assembled by a two-step approach. First, 14,215 contigs were mapped to their Zebra Finch (*Taeniopygia guttata*) orthologues (matching 93.1% of the Zebra Finch reference RNA). This implies that we retrieved the majority of functional genes for *P. w. bulomachus* from a mix of transcriptomes of the cerebrum and liver. An additional 7,508 contigs were mapped to their chicken (*Gallus gallus*) orthologues. A further 465,613 contigs were assembled *de novo* (Table 1).

**Table 1:** **Summary of the numbers of contigs retained in each step when characterizing the transcriptome of *Paradoxornis webbianus bulomachus***

**Step**	**Reference-guided assembly**		***De novo *assembly**	**Total**
	**Zebra Finch**	**Chicken**		
Assembly	14,215	7,508	465,613	487,336
Coverage filtered^1^	10,076	680	57,035	67,791
BLASTed	9,923	528	16,028	26,479
Of which:				
Homology filtered^2^	6,359	31	1,389	7,779
GO annotated	5,426	50	889	6,365

^1^ Contigs filtered with coverage of > 10 X.

^2^ Contigs filtered with similarity (≥ 90%) and alignment length (≥ 200 amino acid residues) with their best matches used to analyze the top-hit species distribution.

**Figure 1:**
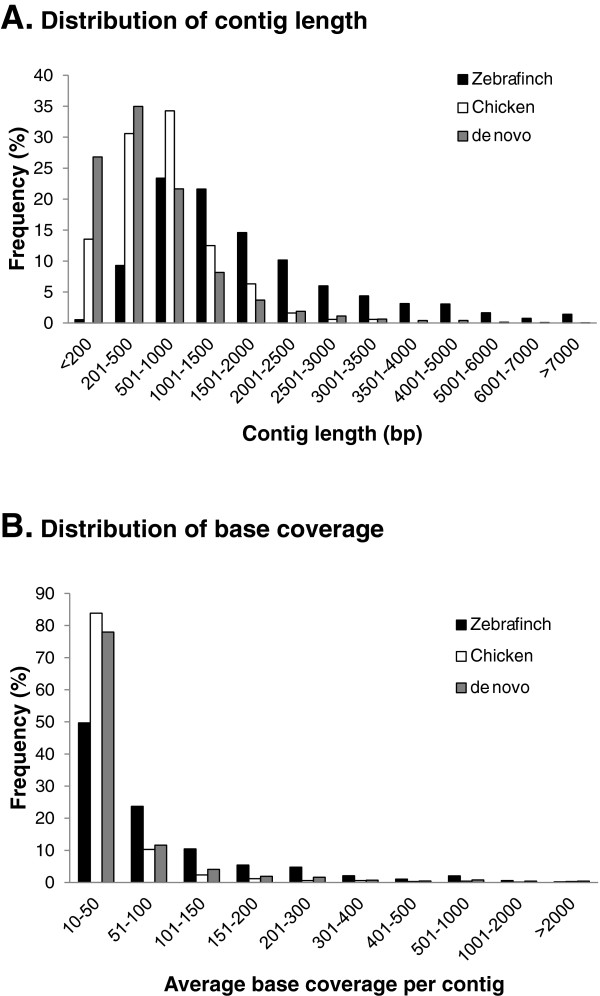
**Distribution of the length and base coverage of the *Paradoxornis webbianus bulomachus *contigs produced through reference and *de novo *assemblies. A.** Distribution of contig length: contig lengths tended to be longer than 1000 bp with reference-based assembly and shorter than 1000 bp with *de novo* assembly. **B.** Distribution of base coverage: base coverage of contigs was generally in the range 10 ~ 100X in both assemblies.

**Figure 2:**
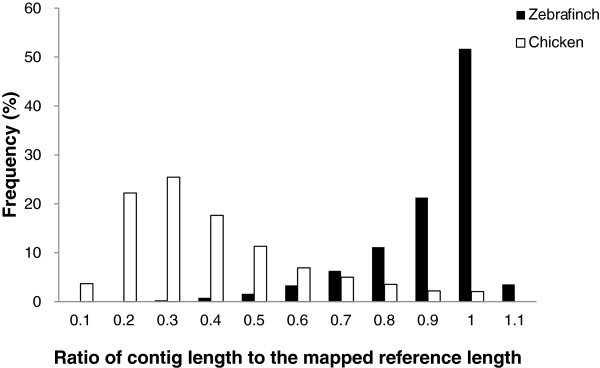
**Distribution of the ratio of the length of a contig to the length of its mapped reference sequence.** Most (71.9%) contigs had a ratio of > 0.9, while only 7.6% of contigs had a ratio of < 0.5.

After filtering out contigs with an average base coverage (sequencing depth) of < 10X, we retained 67,791 (13.9%) unique contigs for subsequent analyses, including 10,076 and 680 contigs from the Zebra Finch and chicken mRNA reference-guided assemblies respectively and 57,035 contigs from *de novo* assembly (Table 1). Distributions of length and base coverage for the retained contigs are shown in Figure 1. In general, contigs assembled under guidance of the Zebra Finch RNA sequences tended to be longer than 1,000 bp (66.8% of the filtered contigs, *N* = 6,731), whereas contigs under chicken RNA guidance and *de novo* assembly tended to be shorter (21.6% and 16.6% of filtered contigs were longer than 1,000 bp, *N* = 533 and 47,594 respectively) (Figure 1A). The large number of contigs produced by *de novo* assembly indicates that there might be some novel genes in the *P. w. bulomachus* genome. The longest contigs in the Zebra Finch and chicken mRNA reference-guided assemblies were 26,364 and 3,489 bp long, respectively, and that for *de novo* assembly was 11,452 bp long. We divided the lengths of the contigs in the reference-guided assemblies by the lengths of their mapped references, and found most to have a ratio of > 0.9 (71.9%; *N* = 7,737). Only 820 contigs had a ratio of < 0.5 (7.6%) (Figure 2). This implies that the deep sequencing power of RNA-seq had efficiently recovered full-length or nearly full-length transcripts. The base coverage of contigs from different assemblies was generally within a range of 10 ~ 100X (Figure 1B). However, contigs with high base coverage of > 100X) occurred more than twice as frequently in the Zebra Finch mRNA-guided assembly (26.6%, *N* = 2,683) than in the chicken mRNA-guided assembly (5.9%, *N* = 40) and *de novo* assembly (10.4%, *N* = 5,953).

Because of similarities of the genomic structure and chromosomal organization among different species of birds [22], we assumed the genome size of *P. w. bulomachus* to be similar to that of the Zebra Finch at approximately 1.2 Gbp [23]. The unique contigs retained in this study covered 64,330,421 bp and represented approximately 5.3% of the *P. w. bulomachus* genome. The reported RNA sequences (including protein-coding sequences and a small proportion of other functional RNAs) of the Zebra Finch and chicken respectively represent approximately 2.4% and 3.5% of their total genomes according to the NCBI database.

### BLAST, top-hit species distribution and GO annotation

All contigs retained after filtering for base coverage (10,076 and 680 retained from the two reference-guided assemblies and 57,035 retained from *de novo* assembly) were BLASTed against the NCBI non-redundant (nr) protein database using Blast2GO [24,25]. Accessions of BLAST hits were then used to retrieve associated sequence descriptions and GO terms.

**Figure 3:**
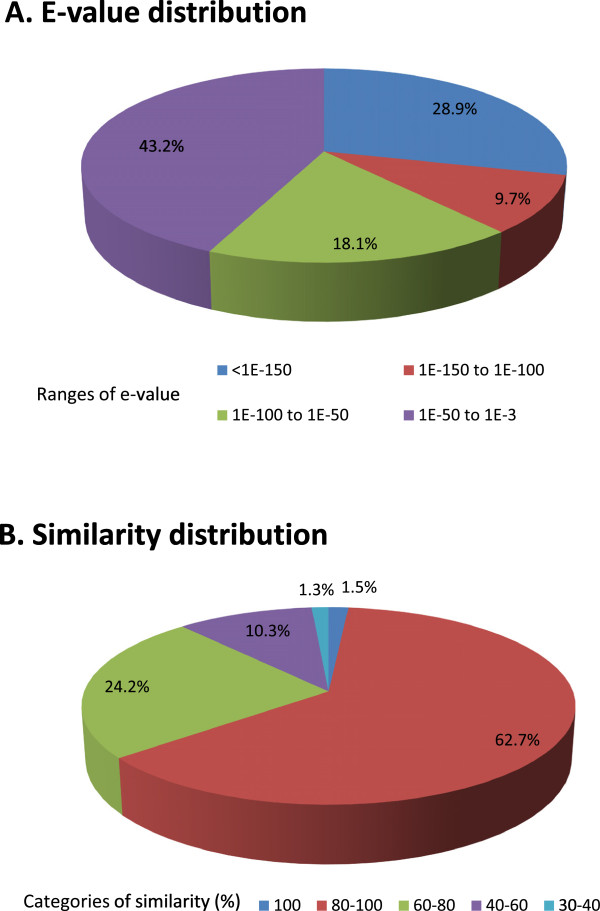
**Distribution of E-values and similarities of *Paradoxornis webbianus bulomachus *contigs with their NCBI nr BLAST matches. A:** E-value distribution of top BLASTx hits of query sequences with a cutoff E-value of < 1.0E^−3^. **B:** Similarity distribution of the top BLASTx hits for each query sequence.

**Figure 4:**
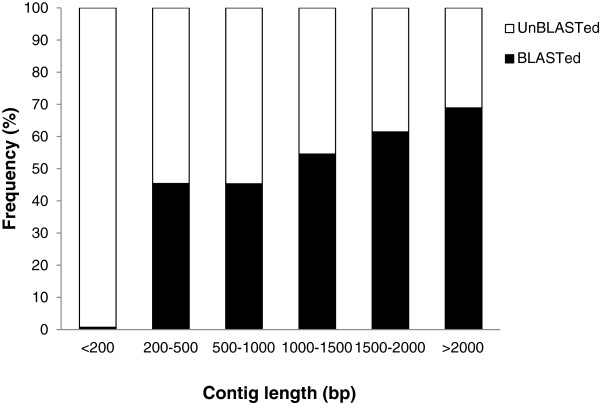
**Effect of query sequence length on the percentage of sequences for which significant matches were found.** The proportion of sequences with matches in the NCBI nr database was higher for longer contigs.

In total, 26,479 contigs (39% of all unique contigs, Table 1) were returned with at least one hit with an E-value of < 1.0E^−3^. Over half of these contigs (57%, *N* = 15,029) had a strong homology with their best hit (i.e., an E-value of < 1.0E^−50^) (Figure 3A). About 64% of the contigs (*N* = 17,000) had a mean similarity with their hits of > 80%, while only 12% of the contigs (*N* = 3,066) had a mean similarity with their hits of < 60% (Figure 3B). Higher proportions of longer contigs gained NCBI nr BLAST matches than shorter contigs: 3,982 or 69% of the 5,778 contigs of > 2,000 bp were matched against the NCBI nr database, while the proportions of matches fell to levels of 0.7% ~ 61% in the other length categories (Figure 4).

To reduce low-quality BLAST matches, we filtered out those with sequence similarities of < 90% and alignment lengths smaller than 200 amino acid residues to retain a total of 7,779 contigs (Table 1). The top-hit species distribution of these filtered contigs is summarized in Table 2. The filtered species list may better reveal the interspecific transcript homology than an unfiltered one because only the best matches with high sequence similarities and long alignment lengths are retained. This is important since, without filtering, similarity introduced merely by sharing short but highly conserved functional domain(s) between sequences of different species can be mistaken for genealogical homology. The vast majority of top matches of filtered *P. w. bulomachus* transcripts were with sequences from avian species (91.55%, *N* = 7,122, with 11 species). Of those species, matches with the Zebra Finch (71.24%, *N* = 5,542) and chicken (15.82%, *N* = 1,231) were most frequent. Top-hit species other than birds were mostly vertebrates including 24 mammals (6.23%, *N* = 485), five reptiles (0.66%, *N* = 51), two amphibians (0.37%, *N* = 29), and four fish (0.18%, *N* = 14). One contig was found to have its top hit to a butterfly species. Another 38 contigs (0.49%) had their best matches to the parasitic protozoan, *Trichomonas vaginalis*, a causative agent of human trichomoniasis. We also found that 36 contigs had their best matches to sequences of four plant species including three crops (maize, rice and sorghum) and one grape species. Since we had a set of relatively stringent criteria to filter out the most unspecific species hits, these matches might reflect a similarity to some longer but conserved protein functional domains. However, it is also possible that they might be due to some aerosol contamination of pollen from unknown plants or contamination from food.

**Table 2:** **Distribution of BLASTx top-hit species of *Paradoxornis webbianus bulomachus *contigs with a similarity of > 90% and an alignment length of > 200 amino acid residues to their best-hit sequences**

**Category Species**	**Reference-guided assembly**			
	**Zebra Finch**	**Chicken**	***De novo *assembly**	**Total**
	**No. of contigs (%)**	**No. of contigs (%)**	**No. of contigs (%)**	**No. of contigs (%)**
**Aves**	**6159 (83.53)**	**27 (0.35)**	**936 (12.03)**	**7122 (91.55)**
*Taeniopygia guttata*	5141 (66.09)		401 (5.15)	5542 (71.24)
*Gallus gallus*	726 (9.33)	20 (0.26)	485 (6.23)	1231 (15.82)
*Meleagris gallopavo*	257 (3.30)	6 (0.08)	38 (0.49)	301 (3.87)
*Zonotrichia albicollis*	20 (0.26)	1 (0.01)	5 (0.06)	26 (0.33)
*Coturnix coturnix*	5 (0.06)		3 (0.04)	7 (0.09)
Other avian species^1^	10 (0.13)		4 (0.05)	15 (0.19)
**Mammals**	**165 (2.12)**	**4 (0.05)**	**316 (4.06)**	**485 (6.23)**
*Monodelphis domestica*	27 (0.35)		67 (0.86)	94 (1.21)
*Homo sapiens*	28 (0.36)	2 (0.03)	26 (0.33)	56 (0.72)
*Mus musculus*	22 (0.28)		33 (0.42)	55 (0.71)
*Ailuropoda melanoleuca*	6 (0.08)		27 (0.35)	33 (0.42)
*Ornithorhynchus anatinus*	10 (0.13)		22 (0.28)	32 (0.41)
*Equus caballus*	5 (0.06)		22 (0.28)	27 (0.35)
*Bos taurus*	5 (0.06)		20 (0.26)	25 (0.32)
*Sus scrofa*	3 (0.04)		16 (0.21)	19 (0.24)
*Macaca mulatta*	5 (0.06)		13 (0.17)	18 (0.23)
*Rattus norvegicus*	4 (0.05)		14 (0.18)	18 (0.23)
*Oryctolagus cuniculus*	2 (0.03)		13 (0.17)	15 (0.19)
*Pongo abelii*	4 (0.05)		9 (0.12)	13 (0.17)
*Callithrix jacchus*	5 (0.06)		8 (0.10)	13 (0.17)
*Loxodonta africana*	7 (0.09)	1 (0.01)	2 (0.03)	10 (0.13)
*Canis familiaris*	1 (0.01)		9 (0.12)	10 (0.13)
*Cricetulus griseus*	5 (0.06)		3 (0.04)	8 (0.10)
Other mammalspecies^2^	26 (0.33)	1 (0.01)	12 (0.15)	39 (0.50)
**Reptiles**	**19 (0.24)**		**32 (0.41)**	**51 (0.66)**
*Anolis carolinensis*	8 (0.23)		28 (0.36)	46 (0.59)
Other reptile species^3^	1 (0.01)		4 (0.05)	5 (0.06)
**Amphibians**	**10 (0.13)**		**19 (0.24)**	**29 (0.37)**
*Xenopus tropicalis*	7 (0.09)		15 (0.19)	22 (0.28)
*Xenopus laevis*	3 (0.04)		4 (0.05)	7 (0.09)
**Fish**	**6 (0.08)**		**8 (0.10)**	**14 (0.18)**
*Danio rerio*			5 (0.06)	5 (0.06)
*Tetraodon nigroviridis*	1 (0.01)		2 (0.03)	3 (0.04)
*Salmo salar*	1 (0.01)		1 (0.01)	2 (0.03)
*Cyprinus carpio*	3 (0.04)			3 (0.04)
**Insect**				
*Danaus plexippus*	1 (0.01)			1 (0.01)
**Protozoa**			**38 (0.49)**	**38 (0.49)**
*Trichomonas vaginalis*			38 (0.49)	38 (0.49)
**Plants**			**36 (0.46)**	**36 (0.46)**
*Zea mays*			33 (0.42)	33 (0.42)
Other plant species^4^			3 (0.04)	3 (0.04)
**Others**^**5**^			**4 (0.05)**	**4 (0.05)**
Total	6359 (87.75)	31 (0.40)	1389 (17.86)	7779 (100)

Totals for each category are in bold.

^1^ Other avian species included *Anas platyrhynchos, Anser anser, Columba livia, Dromaius novaehollandiae, Lonchura striata,* and *Struthio camelus*.

^2^ Other mammal species included *Cavia porcellus, Felis catus, Heterocephalus glaber, Macaca fascicularis, Mustela putorius furo, Nomascus leucogenys, Pan troglodytes,* and *Serinus canaria*.

^3^ Other reptile species included *Alligator mississippiensis, Iguana iguana, Psammobates geometricus*, and *Pseudemys nelson*.

^4^ Other plant species included *Oryza sativa, Sorghum bicolor,* and *Vitis vinifera*.

^5^ Others included sequences of unknown species and synthetic constructs.

**Figure 5:**
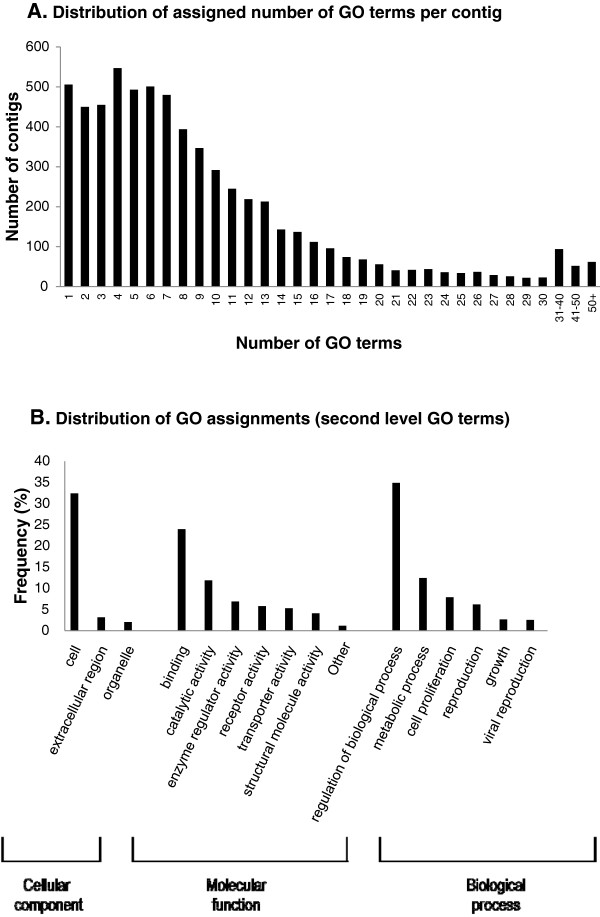
**Functional annotations of *Paradoxornis webbianus bulomachus *contigs. A:** Distribution of the assigned number of gene ontology (GO) terms per contig. The mean level of assigned GO terms was 6.0 ± 1.8 for a total of 60,008 annotated terms. **B:** Distribution of GO assignment (second-level GO terms) in the three main GO categories (cellular component, molecular function and biological process). Counts of terms of < 100 were combined in the figure. ‘Other’ within ‘molecular function’ combines 5 terms including electron carrier (75 counts), antioxidant (31 counts), translation regulator (13 counts), nutrient reservoir (2 counts) activities and protein tag (1 count).

Among all contigs with nr BLASTx hits, 6,365 (24%) were associated with at least one GO term (Table 1). Annotated sequences with a length of > 200 bp (required by NCBI) were deposited in NCBI under accession nos. JR863647 ~ JR870006. In total, 60,008 GO terms were mapped, with a mean of 9.4 ± 9.5 (standard deviation; SD) per contig. The distribution of GO terms mapped per contig is shown in Figure 5A. We used GO-slim to report a simpler functional schema of the *P. w. bulomachus* transcriptome. In total, 122 GO-slim terms (with first-level annotations removed) were assigned, which included biological process (41%, *N* = 50), molecular function (32%, *N* = 39) and cellular component (27%, *N* = 33). The distribution of assignment counts of second-level GO terms within the three major categories is summarized in Figure 5B. Of the six second-level GO terms grouped under ‘biological processes’, those assigned to the largest numbers of contigs were regulation of biological process (*N* = 2,221 counts). Of the 11 second-level GO terms grouped under ‘molecular function’, ‘binding’ (*N* = 1,256 counts) was the most common term assigned to the contigs. In ‘cellular components’ (with three second-level GO terms), most contigs were assigned to ‘cell parts’ (*N* = 2,064 counts).

### Identification of SNPs and SSRs

Polymorphic genetic markers are important tools for detecting population structure, following an organism's demographic history, and answering various important questions in ecology and evolution [24,25]. Herein, we demonstrated that the development of various genetic marker systems can be facilitated by the deep sequencing power of RNA-seq. In this study, 136,984 SNPs were identified from the transcriptome of *P. w. bulomachus* (Table 3) by the SNP detection algorithm implemented in the CLC Genomics Workbench (38,442, 1,633 and 96,909 SNPs were respectively found in the Zebra Finch reference-guided, chicken reference-guided, and *de novo* assemblies). To validate these results, we used the Genome Analysis Toolkit (GATK, [26]) to independently detect SNPs. The GATK detected fewer SNPs (*N* = 17,082) in the Zebra Finch reference-guided assembly than in the CLC Genomics Workbench (*N* = 38,442). This might have resulted from the more stringent setting in the GATK, as a result of which only variants with Phred scores of > 30 (Q30) were reported as SNPs. In total, the two results contained 8,589 overlapping sites in 3,961 contigs with a mean SNP density of 0.91 SNP per thousand base pairs (kbp). These SNPs represent promising SNPs for further study. Overlapping SNPs which have at least 50 bp long of 5′ and 3′ flanking sequences (*N* = 8,455), were deposited in the NCBI dbSNP. They will be publicly available when dbSNP build B137 are released (May, 2012. It may change, please visit dbSNP website for announcement). In these SNP-containing contigs, we found one contig with an extraordinarily high SNP density of 13.04 SNPs/kbp. This contig mapped to the ring finger protein 20 (UGID: 2245576). Assuming it is a single-copy gene in the *P. webbianus* genome, the high variation of the heterozygous genotype indicated that its two haplotypes must have highly diverged and possibly suggests that high balancing selection has acted on it. Validation rates of putative SNPs were reported to be more than twice as high for those expressed as heterozygotes in a single individual than for those expressed as different homozygous alleles in multiple individuals [10]. Screening heterozygous SNP sites of a single individual may, therefore, be a more-efficient way to identify valid SNPs. However, some of the SNPs detected could indeed have been of duplicated genes that share high identity [27].

**Table 3:** **Number of single-nucleotide polymorphisms (SNPs) and simple-sequence repeats (SSRs) identified in the transcriptome of *Paradoxornis webbianus bulomachus***

**Type of marker**	**Reference-guided contigs**		***De novo *contigs**	**Total**
	**Zebra Finch**	**Chicken**		
SNPs	38,442	1,633	96,909	136,984
SSRs				
Di-nucleotide	84	11	815	910
Tri-nucleotide	68	1	290	559
Tetra-nucleotide	16	0	59	75
Total	168	12	1,164	1,344

Using MSATCOMMADER [28], we found 1,344 di-, tri-, or tetra-nucleotide SSR regions with a minimum of seven continuous repeats in all contigs (Table 3). Di-nucleotide repeats were the most abundant SSRs (67.7%, *N* = 910) in the *P. w. bulomachus* transcriptome while tri- and tetra-nucleotide repeats were only present at smaller frequencies (tri-nucleotide SSRs 26.7%, *N* = 359 and tetra-nucleotide SSRs 5.6%, *N* = 75). These SSRs could provide a rich source for further development of a polymorphic marker system for all *P. webbianus* populations.

### Discovering candidate genes for hypoxic and climatic adaptations in the transcriptome of *P. w. bulomachus*

In order to understand how the genetic context of *P. w. bulomachus* populations might have adapted to harsher highland environments, we matched our annotated contigs with 329 candidate genes for high-elevation [5] and climatic adaptations [21]. We found 69 matches: 61 for hypoxia, six for climatic adaptation and two for both hypoxia and climatic adaptations ( Additional file 1: Table S1). Among the 61 hypoxia-related candidate genes, we found five genes (i.e., *PTEN*, *PPARA*, *HOMX2*, *ANGPTL4*, and *EDNRA*) that are associated with the hypoxia-inducible factor (*HIF*) pathway and which were suggested to be under recent positive selection for human high-elevation adaptation [5,29]. We also identified eight genes (*CD36*, *EGFR*, *EPHX2*, *LEPR*, *LPA*, *MAPK1*, *SOD1*, and *TCF7L2*) that may be correlated with thermal adaptation in humans [21]. An additional 40 genes ( Additional file 1: Table S2) that may underpin environmental adaptation were found by searching GO terms of oxygen binding, response to hypoxia, response to cold and response to heat from the *P. w. bulomachus* transcriptome database. In total, we identified 109 genes that can serve as *a priori* candidates to investigate how populations of *P. w. bulomachus* have adapted to diverse ecological environments along an elevational gradient.

## Conclusions

In this study, transcriptome information was obtained from a single individual of the Taiwan endemic subspecies of *P. webbianus*. The Vinous-throated Parrotbill, which occupies broad elevational and latitudinal ranges in East Asia, is an excellent organism to interpret adaptation to diverse environments. The various potential genetic markers obtained can be directly applied to understand how genetic contents can change and diversify under heterogeneous elevational environments in *P. w. bulomachus*, and they can also be applied to understand how the genetic structure may respond to the wide range of latitudes in which *P. webbianus* is found. The causal effect of natural selection on genetic aspects of ecological adaptation can be judged and compared from both vertical and horizontal environmental dimensions in a single species and may provide insights into adaptive evolution in natural populations. This is the first study providing transcriptomic information for species in the species-rich avian superfamily, the Sylvioidea [30]. Therefore, the genomic resources of *P. w. bulomachus* can be used for various ecological and evolutionary studies across different parrotbill species (Paradoxornithidae) [18,31], and can also be extended to the Sylvioidea that comprises more than 1,000 avian species in the world and is the most species-rich avian superfamily in Asia [32].

## Methods

### RNA extraction and sequencing

Fresh cerebrum and liver tissues were removed from an adult male *P. w. bulomachus* collected in Hualien, Taiwan (121′32.54E, 23′54.03 N, elevation: 40 m), and were immediately stored in RNA*later* buffer (QIAGEN) to stabilize the RNA integrity. Tissues were later transferred to a −80°C freezer for long-term storage. Total RNA was isolated using two different commercial kits: RNeasy Lipid Tissue Kit (QIAGEN) for the fat-rich cerebrum and RNeasy Mini Kit (QIAGEN) for the liver. Extractions were performed following the manufacturer’s instructions with an additional DNase digestion procedure using an RNase-Free DNase Set (QIAGEN). The quality and quantity of the total RNA were measured on a 1.2% agarose gel and a NanoDrop spectrophotometer (ND-1000, Thermo). Equal amounts of RNA from each tissue were pooled for RNA-Seq sequencing (mRNA-Seq prep kit, Illumina) and paired-end sequences were obtained from one lane of a HiSeq 2000 Genome Analyzer (Illumina) at BGI (Shenzhen, China). Unfiltered sequences were deposited in the NCBI Short Read Archive under submission no. SRA045008. The original reads were cleaned by removing adaptor sequences and short reads (reads of < 15 bp). Subsequently, reads were trimmed by one nucleotide at both the 5′ and 3′ ends to improve the read quality.

### Transcriptome assembly

We used a two-step strategy described below to assemble the transcriptome of *P. w. bulomachus*, i.e., assembly with guide reference sequences and *de novo* assembly. First, all reads were assembled according to 15,275 RNA reference sequences of Zebra Finch (*T. guttata*, NCBI genome project ID 32405, [23], available at ftp://ftp.ncbi.nlm.nih.gov/genomes/Taeniopygia_guttata/RNA/rna.fa.gz). Subsequently, the remaining reads were assembled according to 19,131 RNA reference sequences of the chicken (*Gallus gallus*, genome build 2.1, available at ftp://ftp.ncbi.nlm.nih.gov/genomes/Gallus_gallus/RNA/rna.fa.gz). Finally, unmatched reads were then *de novo* assembled. All assemblies were processed using CLC Genomics Workbench version 4.8 (CLC Bio) with an option for global alignment that requires exact matches of the 5′- and 3′-end sequences during assembly. For assembly with the reference genomes, parameters of similarity and overlap were set to 0.97 (as suggested in [11]) and 0.70, respectively. For *de novo* assembly, these two parameters were respectively set to 0.97 and 0.65. Thereafter, only contigs with a mean coverage > 10X per base were retained for subsequent analyses.

Transcriptome sequencing efficiency of reference-guided assembly was evaluated in two ways. First, by assuming that *P. w. bulomachus* contigs were assembled to their orthologous Zebra Finch transcripts, we calculated the ratio of the number of contigs to the number of Zebra Finch transcripts (i.e., 15,275) to reveal the efficiency of retrieving *P. w. bulomachus* transcripts. Second, by assuming that no indels occurred between orthologues of the two species, we divided the lengths of *P. w. bulomachus* contigs by the lengths of their Zebra Finch orthologues to show the efficiency of recovering full-length transcripts.

### GO annotation

Following the Blast2GO pipeline [33,34], annotations of transcribed contigs were first BLASTed (BLASTx) against the NCBI non-redundant (nr) protein database, mapped to extract GO terms [35] and then annotated. A standard cutoff E-value of 1.0E^−3^ was use in the BLAST process to collect at most 20 hits for each query sequence. The hits returned by BLASTx were further filtered for matches with significant E-values of < 1.0E^−15^ and with high-scoring segment pairs (HSPs) covering at least 80% of the length of the hit in annotation. The species distribution for matched sequences was analyzed from the best hit of each contig. Instead of using the top-hit species information from all contigs, a subset of information was analyzed to preserve a better genealogical homology between our query sequences and their best matches from the NCBI nr database. Two criteria were applied to retain valid contigs: a similarity of at least 90% and a length alignment of more than 200 amino acid residues to their best matches. A cutoff of 200 amino acid residues was chosen since protein domains have an average length of around 100 amino acid residues [36], and since 75% of proteins with single domains and 87% of those with multiple domains are shorter than 200 amino acid residues [37]. Thus, only retaining contigs with a hit alignment length of more than 200 amino acid residues should have greatly improved the homological aspect of the top-hit species distribution. The Annex function implemented in Blast2GO was performed to improve the annotation density (enrich GO terms for annotating). Finally, an additional GO-Slim procedure (with generic GO-Slim terms) was processed to cut down GOs in order to provide a broad overview of GO annotation of the *P. w. bulomachus* transcriptome.

### SNP and SSR identification

We used the SNP detection algorithm implemented in CLC Genomics Workbench to identify SNPs from this ecologically important species. The quality parameters for SNP detection were set as follows: a window length of 11, a maximum number of gaps and mismatches of 10, an average quality of surrounding bases of 20, and a minimum quality of the central base of 30. To minimize the chance of false positives due to sequencing errors, the minimum variant frequency was set to 35%, and only sites with more than 10X coverage were included for SNP detection. To validate the SNPs called by CLC Genomic Workbench, we used another program, the Genome Analysis Toolkit (GATK) version 1.3.21 [26], to independently detect SNPs in the Zebra Finch RNA reference-guided assembly. We first attempted to conduct the GATK analysis with the CLC output contig alignment. However, because the current version of CLC Genomics Workbench does not keep track of which is the first and second reads of a pair in an assembly (confirmed in consultation with CLC bio global technical support), its exported SAM/BAM files could not be processed in the subsequent GATK procedure. In order to obtain an executable BAM file, we performed an independent alignment with Zebra Finch RNA sequences using the Burrows-Wheeler Aligner (BWA) version 0.6.1 [38]. Duplicates in the mapping results were marked using MarkDuplicates in Picard version 1.57 (http://picard.sourceforge.net/index.html). After running a local realignment and base quality score recalibration, SNP calling was conducted using the GATK UnifiedGenotyper. Variants with a Phred quality score of > 30 (Q30) were exported as SNPs. The SNPs identified by both the CLC Genomics Workbench and the GATK were considered promising SNPs for future investigation.

We used the software, MSATCOMMANDER version 0.8.1 [28], to search for SSRs in the Vinous-throated Parrotbill transcriptome database. Series arrays of di-, tri-, and tetra-nucleotide repeats were searched through all contigs assembled. SSR motifs with more than six repeats were considered to be potential SSR loci for the Vinous-throated Parrotbill.

### Finding candidate genes associated with hypoxia and cold-weather adaptation

We matched the annotated results of the *P. w. bulomachus* transcriptome with an *a priori* list of candidate genes that may be associated with hypoxia [5] and cold-weather adaptation [21] to highland environments. We also searched the annotated results for specific GO terms such as “oxygen binding”, “response to hypoxia”, “response to cold” and “response to heat” to identify additional genes that might also be associated with highland adaptation. Sequences of these candidate gene-associated contigs are in the supplementary information.

## Authors’ contributions

SHL designed the study, and carried out assembly of the transcriptome and identification of the SNPs. JHC annotated the transcriptome and mapped the GO terms to transcripts. RCL conducted RNA extractions, analyzed the assemblies in detail, and validated the SNPs. CFY identified the SSRs from the transcripts. YCH was responsible for fieldwork and tissue collection. JHC, RCL, and SHL shared equal responsibility for preparing the manuscript. All authors read and approved the final manuscript.

## Supplementary Material

Additional file 1: Table S1 Description of candidate genes associated with hypoxia^1^ and climatic adaptation^2^ found from BLASTed contigs of the parrotbill transcriptome. Table S2. Description of candidate genes associated with hypoxia and climatic adaptation by searching GO terms from annotated contigs of the parrotbill transcriptome. Click here for file
