# Supplementary material for: Characterization of the transcriptome of an ecologically important avian species, the Vinous-throated Parrotbill Paradoxornis webbianus bulomachus (Paradoxornithidae; Aves)
Source: BMC Genomics. 2012 Apr 24;13:149. doi: 10.1186/1471-2164-13-149 (PMC3577488; doi:10.1186/1471-2164-13-149)
Supplement: Additional file 1: Table S1 — Description of candidate genes associated with hypoxia1 and climatic adaptation2 found from BLASTed contigs of the parrotbill transcriptome. Table S2. Description of candidate genes associated with hypoxia and climatic adaptation by searching GO terms from annotated contigs of the parrotbill transcriptome. [file 1471-2164-13-149-S1.docx]

Supplemental tables

Table S1. Description of candidate genes associated with hypoxia^1^ and climatic adaptation^2^ found from BLASTed contigs of the parrotbill transcriptome

| Gene (alias) | Gene description | Putative adaptive association | NCBI accessions^3^ |
| --- | --- | --- | --- |
| *ABAT* | 4-aminobutyrate aminotransferase | hypoxia | JR867235 |
| *ADORA1* | adenosine a1 receptor | hypoxia | N.A. |
| *ADORA2A* | adenosine a2a receptor | hypoxia | N.A. |
| *ADORA2B* | adenosine a2b receptor | hypoxia | JR867956 |
| *ADRB2* | beta-2 adrenergic receptor | hypoxia | JR868856 |
| *ANGPTL4* | angiopoietin-like 4 | hypoxia | JR864675 |
| *ARNT* | aryl hydrocarbon receptor nuclear translocator 2 | hypoxia | JR867499 |
| *ARNT2* | aryl hydrocarbon receptor nuclear translocator-like | hypoxia | JR869237 |
| *ATG5* | autophagy protein 5 | hypoxia | JR866991 |
| *ATP1B1* | sodium potassium-transporting atpase subunit beta-1-interacting protein 1-like | hypoxia | N.A. |
| *BCL2L1* | bcl2-like 1 | hypoxia | JR865922 |
| *BNIP3* | bcl2 adenovirus e1b 19kda interacting protein 3-like | hypoxia | JR865944 |
| *CAMK2D* | calcium calmodulin-dependent protein kinase type ii subunit delta-like | hypoxia | JR868961 |
| *CD38* | adp-ribosyl cyclase 1 | hypoxia | JR869405 |
| *CDKN1A* | cyclin-dependent kinase inhibitor 1a | hypoxia | JR869284 |
| *CHRNA4* | neuronal acetylcholine receptor subunit alpha-4 precursor | hypoxia | N.A. |
| *CITED2* | cbp p300-interacting transactivator 2 | hypoxia | N.A. |
| *CYB5R4* | cytochrome b5 reductase 4 | hypoxia | N.A. |
| *CYGB* | cytoglobin | hypoxia | JR866745 |
| *DDIT4* | dna-damage-inducible transcript 4 | hypoxia | JR866618 |
| *ECE1* | endothelin converting enzyme 1 | hypoxia | N.A. |
| *EDNRA* | endothelin receptor type a | hypoxia | JR869299 |
| *EDNRB* | endothelin receptor type b | hypoxia | JR868985 |
| *ERCC3* | tfiih basal transcription factor complex helicase xpb subunit | hypoxia | JR865837 |
| *GCH1* | gtp cyclohydrolase 1 | hypoxia | N.A. |
| *GPX1* | glutathione peroxidase 1 | hypoxia | JR863672 |
| *HAAO* | 3-hydroxyanthranilate -dioxygenase | hypoxia | N.A. |
| *HBB* | hemoglobin beta a subunit | hypoxia | N.A. |
| *HMOX1* | heme oxygenase 1 | hypoxia | JR868307 |
| *HMOX2* | heme oxygenase 2 | hypoxia | JR866688 |
| *HSD11B2* | hydroxysteroid dehydrogenase-like protein 2 | hypoxia | N.A. |
| *HSP90AA1* | heat shock protein hsp 90-alpha | hypoxia | JR868662 |
| *IFNG* | interferon gamma receptor 1 | hypoxia | N.A. |
| *JAG2* | jagged 2 | hypoxia | N.A. |
| *KCNA5* | potassium voltage-gated shaker-related member 5 | hypoxia | JR864206 |
| *NGB* | neuroglobin | hypoxia | JR869302 |
| *NOS1* | nitric oxide synthase 1 | hypoxia | N.A. |
| *PDE5A* | cgmp-inhibited 3 -cyclic phosphodiesterase a | hypoxia | N.A. |
| *PIK3CD* | phosphatidylinositol 3-kinase catalytic delta polypeptide | hypoxia | JR869747 |
| *PIK3R3* | phosphatidylinositol 3-kinase regulatory subunit gamma | hypoxia | JR866733 |
| *PPARA* | peroxisome proliferator-activated receptor alpha | hypoxia | N.A. |
| *PRKAA1* | 5 -amp-activated protein kinase catalytic subunit alpha-1 | hypoxia | JR866974 |
| *PSEN2* | presenilin 2 | hypoxia | JR867605 |
| *PTEN* | phosphatase and tensin homolog | hypoxia | N.A. |
| *RORA* | rar-related orphan receptor a | hypoxia | JR866654 |
| *RORB* | rar-related orphan receptor b | hypoxia | N.A. |
| *SCNN1G* | amiloride-sensitive sodium channel subunit gamma | hypoxia | JR869232 |
| *SMAD3* | mothers against decapentaplegic homolog 3 | hypoxia | N.A. |
| *SMAD4* | mothers against decapentaplegic homolog 4 | hypoxia | JR869298 |
| *SOD3* | extracellular superoxide dismutase | hypoxia | JR865522 |
| *SPR* | sepiapterin reductase | hypoxia | JR869636 |
| *STAT5B* | signal transducer and activator of transcription 5b | hypoxia | JR869006 |
| *TFRC* | transferrin receptor protein 1 | hypoxia | N.A. |
| *TNF* | tumor necrosis factor alpha-induced protein 8-like protein 1 | hypoxia | JR869477 |
| *TXN* | thioredoxin | hypoxia | JR864479 |
| *TXN2* | thioredoxin 2 | hypoxia | JR868248 |
| *UBQLN1* | ubiquilin 1 | hypoxia | N.A. |
| *VEGFA* | vascular endothelial growth factor | hypoxia | JR863982 |
| *VHLL* | von hippel-lindau binding protein 1 | hypoxia | JR867429 |
| *VLDLR* | very low density lipoprotein receptor | hypoxia | JR866248 |
| *XRCC1* | dna repair protein rad51 homolog 1 | hypoxia | JR866951 |
| *CD36* | platelet glycoprotein 4 | climatic adaptation | N.A. |
| *LEPR* | leptin receptor overlapping transcript-like 1 | climatic adaptation | JR867775 |
| *LPA* | apolipoprotein a-i binding protein | climatic adaptation | N.A. |
| *MAPK1* | mitogen-activated protein kinase 1 | climatic adaptation | N.A. |
| *SOD1* | cu zn superoxide dismutase variant 1 | climatic adaptation | N.A. |
| *TCF7L2* | transcription factor 7-like 2 (t-cell hmg-box) | climatic adaptation | JR866368 |
| *EGFR* | epidermal growth factor receptor | hypoxia;climatic adaptation | JR867124 |
| *EPHX2* | epoxide hydrolase microsomal | hypoxia;climatic adaptation | N.A. |

^1^ Candidate gene list of hypoxia was from Simonson *et al.* (2010).

^2^ Candidate gene list of climatic adaptation was from Hancock *et al.* (2008)

^3^ NCBI accession numbers were provided for contigs with successful annotations. Genes without accession numbers can be obtained upon request to the authors.

Table S2. Description of candidate genes associated with hypoxia and climatic adaptation by searching GO terms from annotated contigs of the parrotbill transcriptome

| GO terms | Genes | NCBI accession |
| --- | --- | --- |
| oxygen binding |  |  |
|  | cytochrome family subfamily polypeptide 2 | JR867955 |
|  | cytochrome p450 2u1-like | JR866479 |
|  | hemoglobin alpha a subunit | JR867002 |
|  | superoxide dismutase | JR864561, JR864573 |
|  | superoxide dismutase mitochondrial | JR869160 |
|  | tryptophan-dioxygenase | JR868030 |
| response to hypoxia |  |  |
|  | aquaporin 1 (colton blood group) | JR867045 |
|  | fk506 binding protein 12-rapamycin associated protein 1 | JR865825 |
|  | guanine nucleotide binding protein (g protein) gamma 11 | JR867210 |
|  | guanine nucleotide-binding protein subunit beta-1 | JR868979 |
|  | n-myc downstream regulated gene 1 | JR864155 |
|  | protein kinase c epsilon type | JR867199 |
|  | proto-oncogene tyrosine-protein kinase src | JR865291 |
|  | transcription factor gata-6 | JR866581 |
| response to cold |  |  |
|  | cold inducible rna binding protein | JR867976, JR867984 |
|  | guanosine monophosphate reductase | JR864154 |
|  | plasma membrane calcium-transporting atpase 1 isoform 1 | JR867188 |
|  | plasma membrane calcium-transporting atpase 1 isoform 2 | JR867227 |
| response to heat |  |  |
|  | dnaj homolog subfamily a member 2 | JR866422 |
|  | dnaj homolog subfamily a member mitochondrial isoform 2 | JR865733 |
|  | dnaj homolog subfamily b member 4 | JR864168 |
|  | glutamate-cysteine catalytic subunit | JR866757 |
|  | heat shock 70 kda protein 4l | JR866073 |
|  | heat shock protein 70 | JR868676 |
|  | heat shock protein beta-1 | JR866468 |
|  | hmg box-containing protein 1 | JR865769 |
|  | insulin-like growth factor binding protein 7 | JR868806 |
|  | mitogen-activated protein kinase 8 | JR863687 |
|  | myostatin | JR866641 |
|  | ribosomal protein s6 kinase beta-1 | JR868208 |
|  | somatostatin precursor | JR868819 |
|  | stromal cell-derived factor 1 | JR869354 |
|  | stromal cell-derived factor 1 precursor | JR863938 |
|  | subfamily member 1 | JR866204 |
|  | translation initiation factor eif-2b subunit alpha-like | JR864857 |
|  | translation initiation factor eif-2b subunit beta | JR868640 |
|  | translation initiation factor eif-2b subunit epsilon | JR868827 |
|  | translation initiation factor eif-2b subunit gamma-like | JR866099 |
|  | ubiquitin-like protein smt3 | JR869666 |
|  | v-akt murine thymoma viral oncogene homolog 1 | JR868673 |
